# DEDTI versus IEDTI: efficient and predictive models of drug-target interactions

**DOI:** 10.1038/s41598-023-36438-0

**Published:** 2023-06-07

**Authors:** Arash Zabihian, Faeze Zakaryapour Sayyad, Seyyed Morteza Hashemi, Reza Shami Tanha, Mohsen Hooshmand, Sajjad Gharaghani

**Affiliations:** 1 Laboratory of Bioinformatics and Drug Design (LBD), Institute of Biochemistry and Biophysics, University of Tehran, Tehran, Iran; 2 Department of Computer Science and Information Technology, Institute for Advanced Studies in Basic Sciences (IASBS), Zanjan, Iran; 3 Department of Bioinformatics, Kish International Campus, University of Tehran, Kish, Iran

**Keywords:** Computational biology and bioinformatics, Medical research, Mathematics and computing

## Abstract

Drug repurposing is an active area of research that aims to decrease the cost and time of drug development. Most of those efforts are primarily concerned with the prediction of drug-target interactions. Many evaluation models, from matrix factorization to more cutting-edge deep neural networks, have come to the scene to identify such relations. Some predictive models are devoted to the prediction’s quality, and others are devoted to the efficiency of the predictive models, e.g., embedding generation. In this work, we propose new representations of drugs and targets useful for more prediction and analysis. Using these representations, we propose two inductive, deep network models of IEDTI and DEDTI for drug-target interaction prediction. Both of them use the accumulation of new representations. The IEDTI takes advantage of triplet and maps the input accumulated similarity features into meaningful embedding corresponding vectors. Then, it applies a deep predictive model to each drug-target pair to evaluate their interaction. The DEDTI directly uses the accumulated similarity feature vectors of drugs and targets and applies a predictive model on each pair to identify their interactions. We have done a comprehensive simulation on the DTINet dataset as well as gold standard datasets, and the results show that DEDTI outperforms IEDTI and the state-of-the-art models. In addition, we conduct a docking study on new predicted interactions between two drug-target pairs, and the results confirm acceptable drug-target binding affinity between both predicted pairs.

## Introduction

De novo drug discovery consumes enormous amounts of money and requires a lengthy investigation with no guarantee of success^1^. To overcome these challenges, computational drug discovery methods are increasingly used to identify unknown and hidden drug-target interactions (DTIs) to treat numerous diseases. Computational drug repurposing is a milestone in identifying novel indications for currently marketed drugs against targets of interest. The main idea behind the computational drug repurposing strategies is based on the fact that similar compounds may share similar properties (known as guilt-by-association)^2,3^. Three main approaches exist to perform computational DTIs prediction^4^. The ligand-based approach is the first one and is used when limited information on the target is available. These approaches rely on the concept that similar compounds have similar properties and interact with similar proteins. In other words, the predicted outputs of these approaches completely depend on the number of known ligands per protein, therefore, their reliability may be affected by an insufficient ratio of ligands per protein^5–9^. The second approach is the docking-based approach, which uses the 3D structures of a ligand and a receptor to evaluate the binding affinity between them^10^. The molecular docking approach suffers from the lack of enough 3D structures of ligands and receptors^11^. The third promising approach, the chemogenomic approach, has been defined as the identification and description of all possible molecules that can interact with any therapeutic targets, therefore, enables researchers to address the issue of predicting off-target proteins for therapeutic candidates^12,13^. This approach try to avoid the drawbacks of the aforementioned methods by finding the correlations between the chemical space of ligand and the genomic space of protein^14^. Chemogenomic approaches can be classified into five types: (1) Neighborhood models, (2) Bipartite local models, (3) Network diffusion models, (4) Matrix factorization models, and (5) Feature-based classification models^4^. Matrix factorization is one of the popularly used methods in DTI prediction^15^. Matrix factorization methods^16^ manipulate the DTIs and try to find a latent representation of each drug and each target^16–18^. Despite the many advantages of this method, matrix factorization suffers from several disadvantages. For example, matrix factorization uses the linear inner product of two vectors. Consequently, it is not the best solution to predict the interaction or relation of drug and target. As a result, we suggest avoiding conventional linear matrix factorization in drug repurposing. The authors mentioned the problems of matrix factorization methods in another work^19^.

In the last few years, chemogenomic methods that utilize machine learning to predict DTIs (e.g. deep, transformer, and graph neural network methods) have become widely used. These methods have come to the scene to evade the drawbacks of other DTI prediction approaches. We introduce some of the state-of-the-art chemogenomic methods. NeoDTI^20^ is a graph neural network-based method that utilizes an inductive matrix completion method to predict the DTIs. AutoDTI++^21^ employs an auto-encoder solution in combination with matrix factorization. Because of using matrix factorization, this method suffers from data leakage. HIDTI^22^ generates embeddings of targets and drugs by applying neural networks to their different properties and then concatenates all of them. The concatenation of the processed information of each drug-target pair is fed to a residual neural network to identify their interaction. This method suffers from sparsity as well as incomplete generation of embeddings. MolTrans^23^ belongs to transformer-based methods which borrow concepts from the deep language models. TransDTI^24^ takes advantage of AlphaFold^25^ among other pre-trained embeddings and feeds them to a feed-forward neural network to identify DTIs.

**Figure 1:**
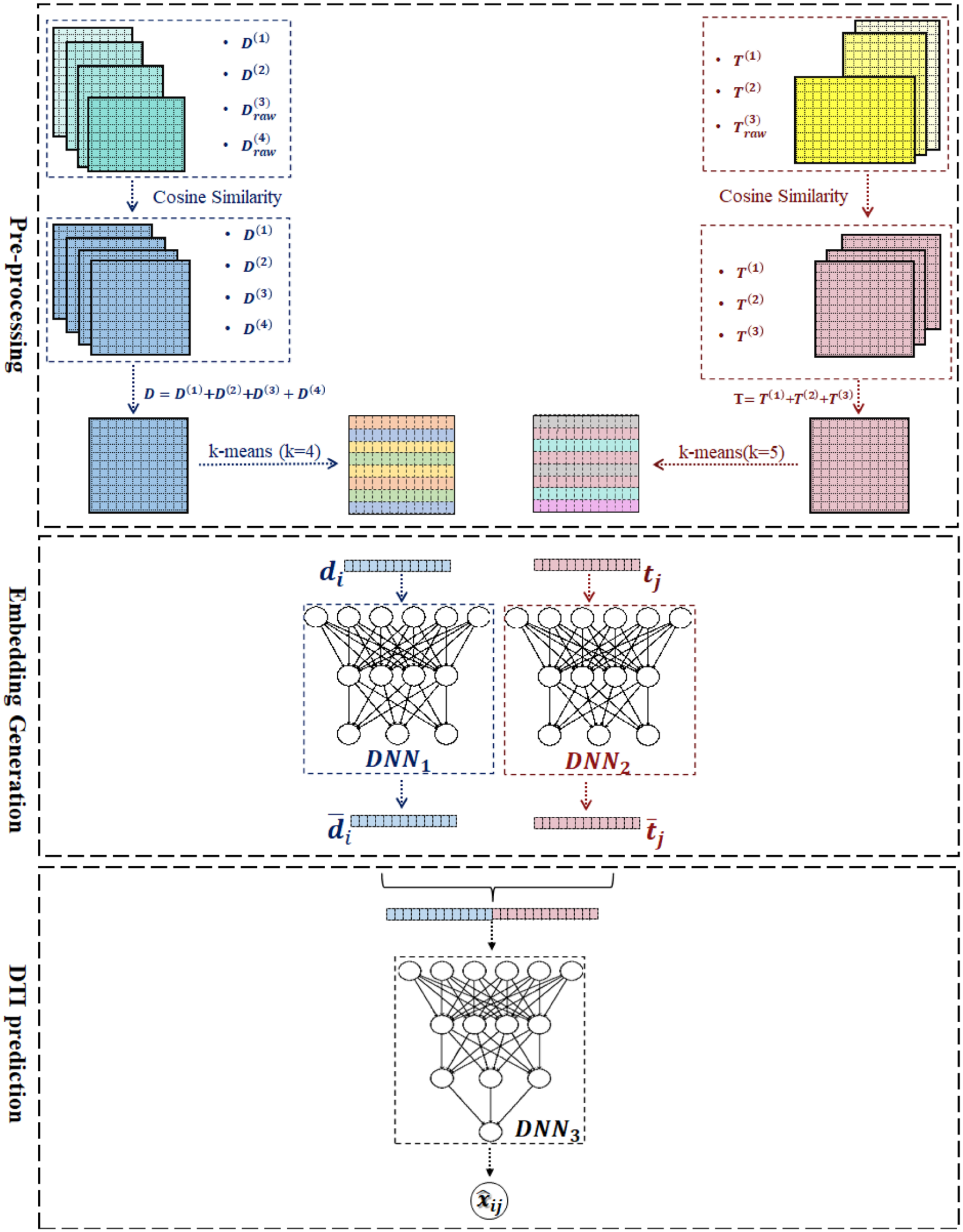
IEDTI’s Framework. It consists of three steps Pre-processing, Embedding generation, and DTI prediction. (I) The first step reads the drug and target matrices. It converts the drug-side effect, drug-disease, and target-disease associations into three similarity matrices. This procedure leads to having four equal-size matrices for drugs and three equal-size matrices for targets. The framework sums up the drug matrices together and sums up the three target matrices as well. It applies k-means to set the same labels for similar drugs. To visualize it, each label is shown in a different color. The same happens for the targets. (II) The framework uses triplet to generate embedding vectors for each drug and target using two DNN modules. (III) It concatenates the embeddings of each drug-target pair and feeds them to the third DNN module to predict interactions.

**Figure 2:**
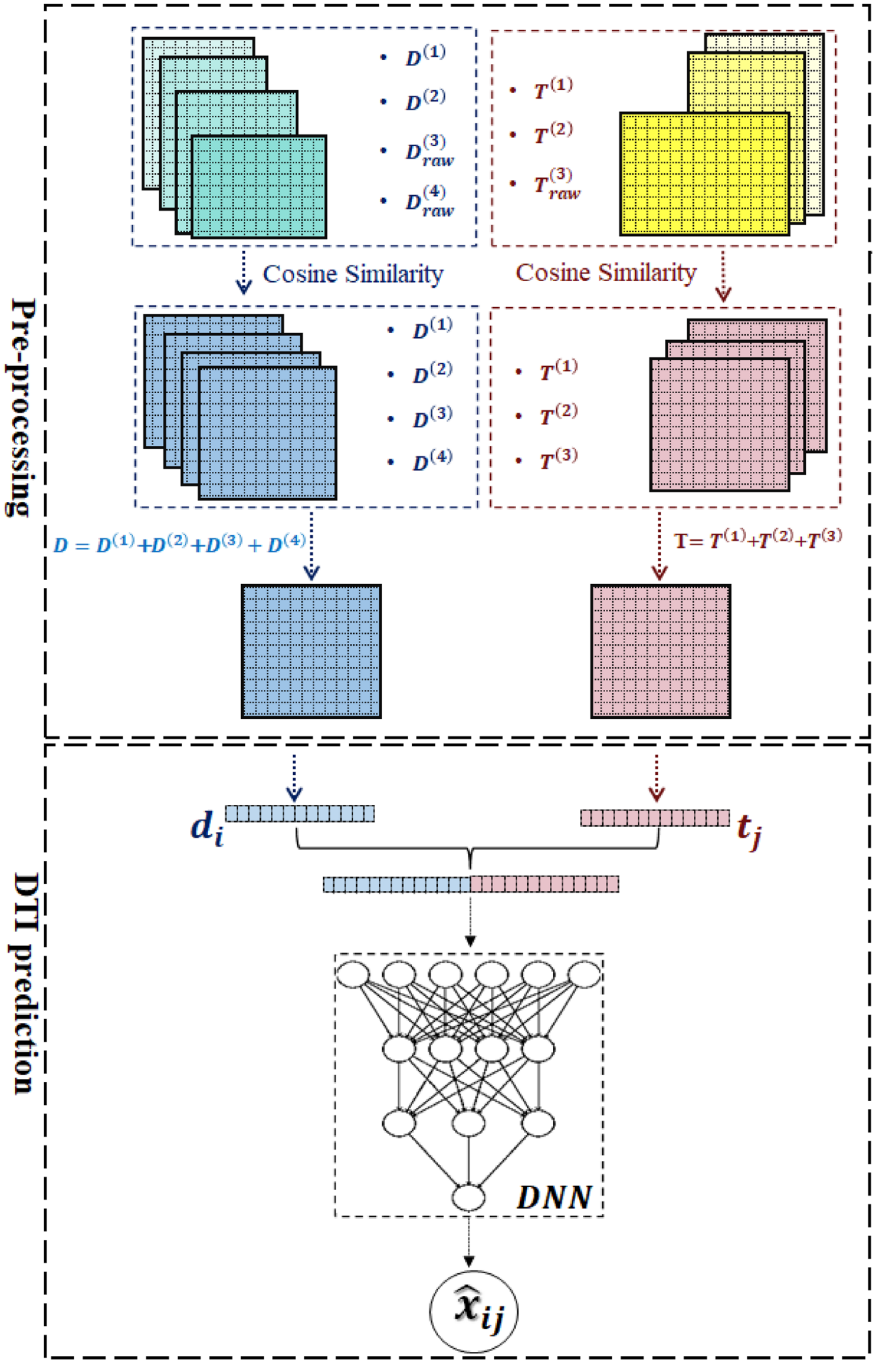
DEDTI’s Framework. This framework consists of two steps, i.e., Pre-processing and DTI prediction. (I) In the pre-processing step, the framework reads the drug and target matrices. It converts the drug-side effect associations, drug-disease associations, and target-disease associations into three similarity matrices. This procedure leads to having four equal-size matrices for drugs and three equal-size matrices for targets. The framework sums up the drug matrices together and sums up the three target matrices as well. (II) The framework concatenates each drug-target pair. It then feeds the concatenations to a deep network module to predict their interactions.

This paper proposes two scenarios for predicting DTIs using a Deep Neural Network (DNN). They vary mainly in the way of modeling the input drug-target pair. We call the first scenario “indirect embedding DTI” or simply *IEDTI* and the second one “direct embedding DTI”, or *DEDTI*. Figures 1 and 2 show proposed frameworks, respectively. We use heterogeneous information, including drug-target interactions, drug-drug interactions, drug-side effect associations, drug-disease associations, target-target interactions, target-disease interactions, and similarities of targets, to predict the DTIs. The "Method" section provides a detailed expression of them.

## Methods

### IEDTI and DEDTI pipelines

IEDTI and DEDTI use drug-target interactions as labels and the remaining information as input to their models. As shown in Fig. 1, the IEDTI has three steps. The first step, pre-processing, involves reading the drug and target matrices and creating their corresponding feature vectors. For drugs, we have two matrices of drug-drug interactions and drug structural similarities. In addition, there are two more matrices of drug-disease and drug-side effect associations. The pre-processing step uses cosine similarity and converts the two latter matrices into similarity matrices. As a result, drugs have four equal-size matrices. We sum them up in the pre-processing step and generate one feature space for drugs. Then, we aim to convert the original feature space to a lower dimensional space. However, the new space needs to preserve the similarities among feature vectors of the original space. To do this, triplet loss is implemented to do meaningful dimension reduction. Triplet loss needs labels of the correlated feature vectors. The original data space does not have any labels. Therefore, the framework applies k-means to the drug vectors, and similar drugs receive the same labels. In other words, we use k-means for sample labeling. This labeling is crucial to prepare the embedding vectors. The same procedure happens for the targets in the pre-processing step.

In the next step—Embedding generation—IEDTI uses two deep network modules ($DNN_1$ and $DNN_2$) for drugs and targets correspondingly. Using $DNN_1$, it maps each drug feature vector into an embedding space. These new representations must have a meaningful interpretation of similar drugs with similar embeddings. The same happens for targets with $DNN_2$.

The last step of IEDTI—DTI prediction—predicts the interaction between each drug-target pair. On the other hand, DEDTI concentrates on DTI prediction exclusively. DEDTI consists of two steps “pre-processing” and “DTI prediction”. It is different from IEDTI by excluding the embedding generation step. We discuss them in more detail as follows.

### Datasets

The datasets were obtained from a previous study on predicting non-homogeneous DTIs^11^ (we call it DTINet dataset). This dataset contains data of 708 drugs from DrugBank (Version 3.0)^26^, 1512 target proteins from the HPRD database (Release 9)^27^, 5603 diseases from the Comparative Toxicogenomics database^28^, and 4192 drug side effects from the SIDER database (Version 2)^29^. Also, there are 1923 known interactions among drugs and targets^30^.

Moreover, we conducted an external validation of Gold standard datasets of Enzyme, GPCR, Ion Channel, and Nuclear Receptor^31^. Table 1 presents all the datasets’ statistics.

**Table 1:** Datasets’ statistics.

Dataset	No of drugs	No of targets	confirmed interactions
DTINet	708	1512	1923
Enzyme	445	664	2926
GPCR	223	95	635
Ion channel	210	204	1476
Nuclear receptor	54	26	90

### Data pre-processing

As mentioned, this study evaluates two scenarios for predicting drug-target interactions-the difference between these two scenarios is rooted in different data pre-processing and manipulation stages. Before diving into the scenarios, we first state the data handling in the datasets. Due to the aim of DTIs’ prediction, this paper addresses interactions among drugs and targets. Eight matrices contain all the information and interaction necessary for our DTI prediction.

- *X*, or Drug-Target interactions with dimension $708\times 1512$ [some studies consider another matrix called Target-Drug matrix. This latter is nothing but the transposition of the former. This paper uses drug-target interaction as the prediction labels, and, therefore, we need just one of them.].
- $D^{(1)}$, or Drug-Drug structural similarities with dimension $708\times 708$.
- $D^{(2)}$, or Drug-Drug interactions with dimension $708\times 708$.
- $D_{raw}^{(3)}$, or Drug-Disease associations with dimension $708\times 5603$.
- $D_{raw}^{(4)}$, or Drug-Side effect associations with dimension $708\times 4192$.
- $T^{(1)}$, or Target-Target interactions with dimension $1512\times 1512$.
- $T^{(2)}$, or Target-Target sequence similarities with dimension $1512\times 1512$.
- $T_{raw}^{(3)}$, or Target-Disease associations with dimension $1512\times 5603$ [It will be clear soon why we have used subscript “*raw*” for some of the matrices $D_{raw}^{(3)}$, $D_{raw}^{(4)}$, and $T_{raw}^{(3)}$. For the moment, these matrices are not similarity matrices.].

It is worth to mention we differentiate the first matrix, *X*, from all other matrices. While we view the other matrices as the input features, *X* is addressed as the DTIs’ prediction labels. The first scenario, **Scenario 1**, deals with embedding generation in addition to the DTI prediction. The second scenario, **Scenario 2**, is exclusively concerned with interaction prediction. In other words, while the former deals with embeddings for further analysis, the latter deals with prediction quality. It is worth mentioning that both methods have the same step of pre-processing.

These two scenarios have a single common step of data pre-processing. Both aim to combine information from drug (and proteins) matrices into a single matrix. The first step transforms the matrices for drugs—$D^{(i)},~1\le i\le 4$—to a single feature matrix, *D*, and for targets—$T^{(j)},~1\le j\le 3$— into a single feature matrix, *T*. $D^{(1)}$ and $D^{(2)}$, both, have the same size of $708\times 708$. To generate the drugs’ feature space, we convert the two other $D^{(3)}$ and $D^{(4)}$ into a space with a size equal to $D^{(1)}$ and $D^{(2)}$. In other words, we get rid of the explicit representation of diseases and side effects from $D^{(3)}$ and $D^{(4)}$, respectively. We produced the similarity matrices of drug-disease, drug-side effect, and target-disease matrices by the “cosine similarity^32^” metric. This type of similarity has been used due to it is scale invariance, directionality awareness, utilization in the recommender systems, and computationally efficient^33,34^.

Assume *O* is a matrix with the size of $o_1\times o_2$. The goal is to compute similarity among its rows. With this aim, we apply the cosine similarity. Its output is a square matrix *R* with the size of $o_1\times o_2$. Thus, the similarity of rows *k* and $\ell ,~1\le k,\ell \le o_1$, $R_{k\ell }$ and is equal to

1 $$\begin{aligned} R_{k\ell }=\frac{O(k,:)\cdot O(\ell ,:)}{||O(k,:)||||O(\ell ,:)||}, \end{aligned}$$

where “$\cdot$” represents inner product of two vectors and $||\cdot ||$ shows the vector’s $\ell ^2$-norm. Equation 1 is applied on all pairs $(k,\ell ), 1\le k,\ell \le o_1$. The resulting matrix *R* has the size of $o_1\times o_1$. $D^{(1)}$, $D^{(2)}$, $T^{(1)}$, and $T^{(2)}$ are already similarity matrices. Thus we apply Equation 1 on the remaining matrices—$D_{raw}^{(3)},$ $D_{raw}^{(4)},$ and $T_{raw}^{(3)},$ and the results are $D^{(3)},$ $D^{(4)},$ and $T^{(3)}.$

Eventually, there are four similarity matrices of drugs $D^{(1)}$, $D^{(2)}$, $D^{(3)}$, and $D^{(4)}$ with the same size of $708\times 708$, and there are three similarity matrices $T^{(1)}$, $T^{(2)}$, and $T^{(3)}$ for target data which the size of them is $1512\times 1512$. These conversions aim to generate feature vectors for drugs as well as targets. We do this by summing up the drug similarity matrices for drugs and target similarity matrices for targets. Thus, the final drug and target similarity matrices (*D* and *T*) are obtained by summation of similarity matrices as follows.

2 $$\begin{aligned} D=D^{(1)}+D^{(2)}+D^{(3)}+D^{(4)} \end{aligned}$$

3 $$\begin{aligned} T=T^{(1)}+T^{(2)}+T^{(3)}, \end{aligned}$$

we consider *D* and *T* as the feature vectors for drugs and targets, respectively. In other words, each row of *D* corresponds to an informative representation of a specific drug. The same applies to the target feature vector *T*. By having *D* and *T*, we can describe the scenarios.

### Formulation of problem

This subsection provides the mathematical formulation of IEDTI and DEDTI.

#### IEDTI formulation

 This scenario aims to produce embeddings and DTI prediction utilizing the input feature vectors *D* and *T*. It generates an embedding for each drug ${{\textbf {d}}}_i=D(i,:);~1\le i\le m$ and each target ${{\textbf {t}}}_j=T(j,:);~1\le j\le n$. The embeddings of ${{\textbf {d}}}_i$ and ${{\textbf {t}}}_j$ are $\bar{{{\textbf {d}}}}_i$ and $\bar{{{\textbf {t}}}}_j$, respectively. These new representations occupy smaller spaces, leading to faster and more efficient computation. In addition, they have meaning, i.e., similar vectors have similar embedding representations, and different ones have dissimilar representations. Then, it predicts the DTIs. We first explain the way of embedding generation. We start with describing the production of drugs’ embeddings. Each drug ${{\textbf {d}}}_i$ of the *D* matrix is mapped into a new representation space and is shown by $\bar{{{\textbf {d}}}}_i$. In other words, those drugs are transformed into a new domain by meeting the “significant property” of similar pair of vectors having similar pair of embedding vectors and vice versa. Thus, we look for a function, i.e., $g_1$, where it converts each ${{\textbf {d}}}_i$ of *D* to an embedding vector with the property of similar ones must have similar embedding vectors and dissimilar ones must have dissimilar embedding, or formally:

$$\begin{aligned} \forall i \in \{1,\cdots ,m\}, ~\exists k,\ell \in \{1,\cdots ,m\}, k\ne \ell , i\ne k, i\ne \ell ,{\left\{ \begin{array}{ll} {{\textbf {d}}}_i=D(i,:) {{\textbf {d}}}_k=D(k,:)\\ {{\textbf {d}}}_l=D(\ell ,:) \end{array}\right. } s.t.~\\ \exists ~\tau _{D}\in {\mathbb {R}}: {\left\{ \begin{array}{ll} dist_D({{\textbf {d}}}_i,{{\textbf {d}}}_k) < \tau _D\\ dist_D({{\textbf {d}}}_i,{{\textbf {d}}}_{\ell }) > \tau _D \end{array}\right. } \end{aligned}$$

4 $$\begin{aligned} \Longrightarrow {\left\{ \begin{array}{ll} \bar{{{\textbf {d}}}}_i=g_1({{\textbf {d}}}_i)\\ \bar{{{\textbf {d}}}}_k=g_1({{\textbf {d}}}_k) \bar{{{\textbf {d}}}}_{\ell }=g_1({{\textbf {d}}}_{\ell }) \end{array}\right. } s.t.\nonumber \\ \exists ~\tau _{\bar{D}}\in {\mathbb {R}}: {\left\{ \begin{array}{ll} dist_{\bar{D}}(\bar{{{\textbf {d}}}}_i,\bar{{{\textbf {d}}}}_k) < \tau _{\bar{D}}\\ dist_{\bar{D}}(\bar{{{\textbf {d}}}}_i,\bar{{{\textbf {d}}}}_{\ell }) > \tau _{\bar{D}} \end{array}\right. }, \end{aligned}$$

where $\tau _D\in {\mathbb {R}}^+$ and $\tau _{\bar{D}}\in {\mathbb {R}}^+$ are comparison thresholds for drugs’ original representations and embedded representations, respectively. It is worth noting that ${{\textbf {d}}}_i\in {\mathbb {R}}^m$ and $\bar{{{\textbf {d}}}}_i\in {\mathbb {R}}^{f_1}$, where $f_1\ll m$. Two functions $dist_D$ and $dist_{\bar{D}}$ are, respectively ${\mathbb {R}}^m\times {\mathbb {R}}^m\rightarrow {\mathbb {R}}^+$ and ${\mathbb {R}}^{f_1}\times {\mathbb {R}}^{f_1}\rightarrow {\mathbb {R}}^+$ functions, used to measure the similarity among vectors in *D* and their embedding vectors. Distance function can be any legitimate function that discriminates dissimilar vectors and lumps similar vectors in the embedding representation coordinate. The same condition applies to the members of the target similarity matrix (*T*). So, we look for a function $g_2$ with similar conditions on ${{\textbf {t}}}_j$, or formally:

$$\begin{aligned} \forall j \in \{1,\cdots ,n\}, ~\exists k,\ell \in \{1,\cdots ,n\}, k\ne \ell , j\ne k, j\ne \ell ,{\left\{ \begin{array}{ll} {{\textbf {t}}}_j=T(j,:)\\ {{\textbf {t}}}_k=T(k,:)\\ {{\textbf {t}}}_l=T(\ell ,:) \end{array}\right. } s.t.\\ \exists \tau _T\in {\mathbb {R}}: {\left\{ \begin{array}{ll} dist_T({{\textbf {t}}}_j,{{\textbf {t}}}_k) < \tau _T\\ dist_T({{\textbf {t}}}_j,{{\textbf {t}}}_{\ell }) > \tau _T \end{array}\right. } \end{aligned}$$

5 $$\begin{aligned} \Longrightarrow {\left\{ \begin{array}{ll} \bar{{{\textbf {t}}}}_j=g_2({{\textbf {t}}}_j)\\ \bar{{{\textbf {t}}}}_k=g_2({{\textbf {t}}}_k)\\ \bar{{{\textbf {t}}}}_{\ell }=g_2({{\textbf {t}}}_{\ell }) \end{array}\right. } s.t.\nonumber \\ \exists \tau _{\bar{T}}\in {\mathbb {R}}: {\left\{ \begin{array}{ll} dist_{\bar{T}}(\bar{{{\textbf {t}}}}_j,\bar{{{\textbf {t}}}}_k) < \tau _{\bar{T}}\\ dist_{\bar{T}}(\bar{{{\textbf {t}}}}_j,\bar{{{\textbf {t}}}}_{\ell }) > \tau _{\bar{T}} \end{array}\right. } \end{aligned},$$

where $\tau _T\in {\mathbb {R}}^+$ and $\tau _{\bar{T}}\in {\mathbb {R}}^+$ are comparison thresholds for targets’ original representations ${{\textbf {t}}}_i\in {\mathbb {R}}^n$ and embedded representations $\bar{{{\textbf {t}}}}_i\in {\mathbb {R}}^{f_2}$, where $f_2\ll n$, respectively. Each row, $\bar{{{\textbf {d}}}}_i$ and $\bar{{{\textbf {t}}}}_j$ are embedding vectors in a new domain of its corresponding rows, ${{\textbf {d}}}_i$ and ${{\textbf {t}}}_j$, in target and drug similarity matrices, respectively. Similar to $dist_D$ and $dist_{\bar{D}}$, two other functions $dist_T$ and $dist_{\bar{T}}$ are respectively ${\mathbb {R}}^n\times {\mathbb {R}}^n\rightarrow {\mathbb {R}}^+$ and ${\mathbb {R}}^{f_2}\times {\mathbb {R}}^{f_2}\rightarrow {\mathbb {R}}^+$ functions which are used to measure the similarity among vectors in *T* and their embedding vectors. The $\bar{{{\textbf {d}}}}_i,~1\le i \le m$ and $\bar{{{\textbf {t}}}}_j,~1\le j \le n$ are the first type of **Scenario 1** output. The next type is the prediction of interaction between drug-target pairs. To do this, it uses each pair of $\bar{{{\textbf {d}}}}_i$ and $\bar{{{\textbf {t}}}}_j$, and calls a function $g_3:{\mathbb {R}}^{f_1}\times {\mathbb {R}}^{f_2}\rightarrow {\mathbb {R}}$ where $g_3(\bar{{{\textbf {d}}}}_i,\bar{{{\textbf {t}}}}_j)\approx x_{ij}$. We formally define it as follows:

$$\begin{aligned} \forall i \in \{1,\cdots ,m\},~\forall j \in \{1,\cdots ,n\},~x_{ij}=X(i,j), ~{\left\{ \begin{array}{ll} {{\textbf {d}}}_i=D(i,:)\longrightarrow \bar{{{\textbf {d}}}}_i=g_1({{\textbf {d}}}_i)\\ {{\textbf {t}}}_j=T(j,:)\longrightarrow \bar{{{\textbf {t}}}}_j=g_2({{\textbf {t}}}_j) \end{array}\right. },~\exists \varepsilon \end{aligned}$$

6 $$\begin{aligned} \Longrightarrow \nonumber \\ \bar{x}_{ij}=g_3(\bar{{{\textbf {d}}}}_i,\bar{{{\textbf {t}}}}_j),~~s.t.~~dist_{DT}(\bar{x}_{ij},x_{ij})< \varepsilon \end{aligned}$$

Notably, the above explanations are the conceptual formalization of our proposal. The parameters $\tau _D$ and $\tau _T$ are handled using clustering and DNN modules. In other words, we will address these three goals with a *DNN* solution. Our proposed *DNN* is formed of three modules ($DNN_1,~DNN_2,~DNN_3$), and each of them models one of the functions $\{g_1,g_2,g_3\}$. The first module ($DNN_1$) is to compute the embedding of the drug similarity vectors (*D*). Its input vectors are the rows (${{\textbf {d}}}_i$) of *D*, and its output is the new representation of each row, $\bar{{{\textbf {d}}}}_i$. The second module ($DNN_2$) is for acquiring the target embedding vectors ($\bar{{{\textbf {t}}}}_j$). Its input vectors are from the rows (${{\textbf {t}}}_j$) of the target similarity matrix. These two DNN modules act as triplet methods. Finally, the third module ($DNN_3$), by having the inputs in the form of concatenated vectors $(\bar{{{\textbf {d}}}}_i,\bar{{{\textbf {t}}}}_j)$, predicts the interactions between entities of *D* and *T* matrices. The next section provides the structure of the designed *DNN* in more detail.

#### DEDTI formulation

 This scenario directly focuses on DTIs prediction. To do this, **Scenario 2** consists of two steps. The first step is to define the feature vector necessary for DTIs prediction. It utilizes the vectors of *D* and *T* to generate the feature vector required for the prediction. In other words, each feature vectors are available drug-target pair. Each feature vector ${{\textbf {z}}}$ is derived from the ${{\textbf {d}}}_i=D(i,:);~1\le i\le m$ with target ${{\textbf {t}}}_j=T(j,:);~1\le j\le n$, or ${{\textbf {z}}}=({{\textbf {d}}}, {{\textbf {t}}})$, and ${{\textbf {z}}}\in {\mathbb {R}}^{m+n}$. The next step is predicting the interaction between each given drug-target pair. We show both steps as follows.

$$\begin{aligned} \forall i \in \{1,\cdots ,m\}:~{{\textbf {d}}}_i=D(i,:),~\forall j \in \{1,\cdots ,n\}:~{{\textbf {t}}}_j=T(j,:)\Longrightarrow {{\textbf {z}}}_{i,j}=[{{\textbf {d}}}\mathbin \Vert {{\textbf {t}}}] \end{aligned}$$

7 $$\begin{aligned} \forall i \in \{1,\cdots ,m\},~\forall j \in \{1,\cdots ,n\}:~x_{ij}=X(i,j),~{{\textbf {z}}}_{i,j}=[{{\textbf {d}}}\mathbin \Vert {{\textbf {t}}}],~\exists \varepsilon \Longrightarrow \bar{x}_{ij}=g({{\textbf {z}}}_{i,j}),~~s.t.~~dist_{DT}(\bar{x}_{ij},x_{ij})< \varepsilon \end{aligned}$$

### Models’ architecture

This subsection provides the deep architecture of IEDTI and DEDTI. We describe them one by one as follows.

#### IEDTI architecture

This subsection provides the deep architecture of IEDTI. We describe it in three different modules as follows.

1. *First module of Deep Neural Network* The first module ($DNN_1$) gets the ${{\textbf {d}}}_i=D(i,:),\forall i \in \{1,~\cdots ,m\}$ as input and returns the corresponding embedding vector for each of them. As mentioned earlier, the similarity and dissimilarity among targets should also be kept among their corresponding embedding vectors. In other words, if two vectors are similar in the main space, their transformation should be similar in the embedding space. To keep similarities in the embedding space, we take advantage of the idea that Bordes et al. have introduced^35^. However, we have changed the objective function. Let’s assume that for each ${{\textbf {d}}}_i$, we can find the “set” of its similar vectors in *D*. We call it $Smlr_{{{\textbf {d}}}_i}$ . On the other hand, each ${{\textbf {d}}}_i$ has dissimilarities or fewer similarities with the remaining vectors of *D*. Using these two sets of similar ones as well as dissimilar ones for each ${{\textbf {d}}}_i$; we compute its representation $\bar{{{\textbf {d}}}}_i$. Their formulation can be:
  8 $$\begin{aligned} \forall i \in \{1,\cdots ,m\}, {{\textbf {d}}}_i\in D, \tau _D \in {\mathbb {R}}: Smlr_{{{\textbf {d}}}_i}=\{\exists k \in \{1,\cdots ,m\}, {{\textbf {d}}}_k\in D, k\ne i, dist_D({{\textbf {d}}}_i,{{\textbf {d}}}_k)<\tau _D \} \end{aligned}$$
  Having this set and its complement set for each ${{\textbf {d}}}_i\in D$, we define the below objective function:
  9 $$\begin{aligned} {\mathscr {L}}_d=\sum _{i=1}^{m}\sum _{k\in Smlr_{{{\textbf {d}}}_i}}\sum _{\ell \notin Smlr_{{{\textbf {d}}}_i}} \max \left( 0, \gamma + dist_{{\bar{D}}}(\bar{{{\textbf {d}}}}_i,\bar{{{\textbf {d}}}_k})-dist_{\bar{D}}(\bar{{{\textbf {d}}}}_i,\bar{{{\textbf {d}}}}_\ell )\right) \end{aligned}$$
  It is notable that the set $Smlr_{{{\textbf {d}}}_i}$ is defined based on $dist_D$ and ${{\textbf {d}}}$, but ${\mathscr {L}}_d$ is based on $dist_{\bar{D}}$ and $\bar{{{\textbf {d}}}}$. The similar vectors should have a smaller distance, and the dissimilar vectors must have a longer distance. If the model works properly, ${\mathscr {L}}_d$ must be close to zero. Thus, the objective of $DNN_1$ is to minimize the cost function ${\mathscr {L}}_d$. The parameter $\gamma$ is a margin hyperparameter for tuning the objective function. This function is called a triplet. To do this, we can have several layers of neural networks. The number of input layer neurons must be equal to *m* (the length of ${{\textbf {d}}}_i$). It is also necessary for the number of neurons of the output layer to be equal to $f_1$ (the length of $\bar{{{\textbf {d}}}}_i$). It is necessary to have meaningful embeddings. In other words, similar drugs must have similar representations in the embedding space. This aim requires defining a similarity among the original representation of drugs. To this end, we use the k-means algorithm and apply it to the drug vectors and define sets of similar drugs. Using this clustering, $DNN_1$ computes similar embeddings for the drugs of each set. As mentioned above, we applied the k-means method to put similar drugs (and similar proteins) in the same clusters. Then, we obtain a new representation using a semi-hard triplet loss function. This approach leads to having a shorter distance between every two members in a cluster and a wider gap between each pair of clusters. These clusters act as labels, and the loss function uses them to produce meaningful embeddings. Figure 4 shows t-SNE representations of drugs and targets before and after applying triplet. They show the power of k-means’ representation as well as applying triplet embedding vectors. We chose the number of clusters in a way that the clusters have to be roughly equal. Thus, we examined 2 to 64 as the number of clusters for drugs, and 4 is the best possible number of drug clusters. Figure 4a illustrates drugs’ k-means representations. Figure 4b is those drugs’ separation in the embedding coordinate. Comparing two figures shows the discriminating power of the triplet. The same went for the targets; the best number of clusters was 5. Figure 4c shows the result of applying k-means on targets. Finally, Fig. 4d visualizes the final targets’ embeddings.
2. *Second module of Deep Neural Network* The second module ($DNN_2$) works like its sibling $DNN_1$. The difference is that while $DNN_1$ calculates embeddings of ${{\textbf {d}}}_i\in D,~i \in \{1,\cdots ,m\}$, $DNN_2$ calculates ${{\textbf {t}}}_j\in T,~j \in \{1,\cdots ,n\}$. For each ${{\textbf {t}}}_j$, we define sets of similar vectors as well:
  10 $$\begin{aligned} \forall j \in \{1,\cdots ,n\}, {{\textbf {t}}}_j\in T, \tau _T \in {\mathbb {R}}: Smlr_{{{\textbf {t}}}_j}=\{\exists k \in \{1,\cdots ,n\}, {{\textbf {t}}}_k\in T, k\ne j, dist_T({{\textbf {t}}}_j,{{\textbf {t}}}_k)<\tau _T \} \end{aligned}$$
  Having the similarity set of each ${{\textbf {t}}}_i\in T$ and its corresponding complement, we define the below objective function:
  11 $$\begin{aligned} {\mathscr {L}}_t=\sum _{j=1}^{n}\sum _{k\in Smlr_{{{\textbf {t}}}_j}}\sum _{\ell \notin Smlr_{{{\textbf {t}}}_j}} \max \left( 0, \gamma + dist_{{\bar{T}}}(\bar{{{\textbf {t}}}_j},\bar{{{\textbf {t}}}_k})- dist_{{\bar{T}}}(\bar{{{\textbf {t}}}_j},\bar{{{\textbf {t}}}_\ell })\right) \end{aligned}$$
  As we have mentioned for ${{\textbf {d}}}$, the distance between similar and dissimilar vectors must work the same for ${{\textbf {t}}}$ as well. If the model works appropriately, ${\mathscr {L}}_t$ must be close to zero, and the objective of $DNN_2$ is to minimize the cost function ${\mathscr {L}}_t$. For this aim, the first layer of $DNN_2$ should have *n* neurons, and the output layer of $DNN_2$ needs to have $f_2$ neurons. In harmony with the previous subsection, we apply the k-means algorithm to locate the set of similar targets.
3. *Third module of Deep Neural Network* The third module of the neural network $DNN_3$ is in charge of DTI prediction. The input of the $DNN_3$ is the embedding representations of the drug and target from $DNN_1$ and $DNN_2$—the output of $DNN_1$ is the vector $\bar{{{\textbf {d}}}}_{f_1\times 1}$, and the output of $DNN_2$ is the vector $\bar{{{\textbf {t}}}}_{f_2\times 1}$. The input format of $DNN_3$ is the concatenation of $\bar{{{\textbf {d}}}}$ and $\bar{{{\textbf {t}}}}$, or $[\bar{{{\textbf {d}}}}^T \bar{{{\textbf {t}}}}^T]^T$. So, the number of input layer neurons of $DNN_3$ is equal to $f_1+f_2$. As mentioned above, the role of the third section is the calculation of the amount of interaction between $\forall i \in \{1,\cdots ,m\}: {{\textbf {d}}}_i\in D$ and $\forall j \in \{1,\cdots ,n\}: {{\textbf {t}}}_j\in T$, or $x_{ij}$. The output layer has one neuron, an approximation $x_{ij}$. Formally, the objective of $DNN_3$ is
  12 $$\begin{aligned} \min \sum _{i=1}^{m}\sum _{j=1}^{n}[x_{ij}-g_3(\bar{{{\textbf {d}}}}_i,\bar{{{\textbf {t}}}}_j)] \end{aligned}$$
  Because $\bar{{{\textbf {d}}}}_i$ and $\bar{{{\textbf {t}}}}_j$ are acquired from $DNN_1$ and $DNN_2$, we can rewrite the objective function as
  13 $$\begin{aligned} \min \sum _{i=1}^{m}\sum _{j=1}^{n}[x_{ij}-g_3\left( g_1({{\textbf {d}}}_i)\mathbin \Vert g_2({{\textbf {t}}}_j)\right) ] \end{aligned},$$
  where $\mathbin \Vert$ shows the concatenations of two vectors. It is necessary to mention that all $DNN_1$, $DNN_2$, and $DNN_3$ can have several hidden layers. We discuss this more in the “implementation” and “discussion” sections. Figure 1 shows the general structure of the first proposed scenario.

It is notable that IEDTI model is not an end-to-end model. Therefore, the error propagation is not an end-to-end process. and each module has its own error propagation.

#### DEDTI architecture

 The deep network of the second scenario is similar to the first one. The only difference is in the input vector of the network. Its input vector is the concatenation of each ${{\textbf {d}}}_i$ and ${{\textbf {t}}}_j$. Formally,

$$\begin{aligned} \min \sum _{i=1}^{m}\sum _{j=1}^{n}[x_{ij}-g({{\textbf {d}}}_i,{{\textbf {t}}}_j)], \quad \forall i \in \{1,\cdots ,m\}: {{\textbf {d}}}_i\in D,~ \forall j \in \{1,\cdots ,n\}: {{\textbf {t}}}_j\in T,~ x_{ij}=X(i,j) \end{aligned},$$

or more precisely, it is

14 $$\begin{aligned} \min \sum _{i=1}^{m}\sum _{j=1}^{n}[x_{ij}-g\left( {{\textbf {d}}}_i\mathbin \Vert {{\textbf {t}}}_j\right) ] \end{aligned}$$

The input layer’s required neurons equal $m+n$, and the last layer contains a single neuron to predict each DTI.

### Implementation

In both described scenarios, we implemented ten-fold cross-validation to provide accurate information about our algorithm performance. To tune the parameters, we have tested the results with the suggestion from the previous studies on the subject of deep Learning and DTI prediction. The results show the parameters perform well in this work.

- *DEDTI model* Our first model takes the concatenation of *i*th protein and *j*th drug vector representations, $c_{ij}$, as input. Therefore, the input shape is (2220, 1) as we have 708 drugs and 1512 targets. Then, it passes input, $c_{ij}$, to four consecutive Conv1D layers with *Relu* activation function, where each is followed by batch normalization and dropout 0.5. Next, we use a dense layer after a flattened layer, followed by a dropout of 0.5. Finally, a dense layer with a sigmoid activation function predicts the interaction between the drug and protein. We compiled our model with Adam optimizer and Binary cross entropy loss function. The interaction is binary-valued. Zero shows no interaction, and one represents valid interaction. We also used the initial bias technique in our final dense layer to consider the imbalance dataset property. Our initial bias is as follows:
  15 $$\begin{aligned} initial~bias = \log \left(\frac{positive~samples}{negative~samples}\right) \end{aligned}$$
  In this model, we set the batch size to 1024 in the training phase.
- *IEDTI model* Our prediction phase in the triplet model is the same as our first model. However, here we have two extra steps. First, we use k-means on drugs and proteins separately to find different clusters in them. Then we obtain new representations for them by using semi-hard triplet loss. Our new vector representation for drugs and proteins has a size equal to 256. After that, we feed their concatenations to our prediction phase, similar to our previous model. However, the input shape in this scenario is (512). As the input shape here is more petite than the previous model, we set our batch size to 64 for this one.

### Performance evaluation metrics

We use ten-fold cross-validation to assess the performance of the models. We used different metrics such as AUC-ROC, AUPR, F1-score, and MCC to evaluate the methods. AUC-ROC is not proper for imbalance. Thus, we used the other evaluation metrics to cover the case of imbalanced data. We compute the sensitivity(recall), specificity, precision, and F1-score metrics based on the following equations.

16 $$\begin{aligned} Sensitivity=\frac{TP}{TP+FN} \end{aligned}$$

17 $$\begin{aligned} Specificity=\frac{TN}{TN+FP} \end{aligned}$$

18 $$\begin{aligned} Precision=\frac{TP}{TP+FP} \end{aligned}$$

19 $$\begin{aligned} F1-score=2\times \frac{ precision\times recall }{precision+recall} \end{aligned}$$

While F1-score is used for imbalanced data evaluation, we considered MCC due to its advantages in binary classification^36^. Its equation is as follows.

20 $$\begin{aligned} MCC=\frac{ TP\times TN - FP\times FN }{\sqrt{(TP+FP)(TP+FN)(TN+FP)(TN+FN)} } \end{aligned}$$

### Complexity analysis

The parameter *m* shows the number of drugs, and the number of targets *n* represents the number of targets, the number of diseases is $n_{di}$, and the number of side effects is $n_{se}$. We assume there are $e_{emb}$ epochs necessary for the generation of secondary representations of drugs and targets, and each epoch time is equal to $T_{e}$ for both drug and target. For simplicity, we have assumed no difference in conversion time between the drug and the target. Lastly, we assume the number of epochs in the predictive model is equal to $e_{p}$, and the time interval of each epoch is equal to $T_{p}$.

DEDTI and IEDTI need to compute the primary representation of each drug and each protein. Two similarity matrices for drugs are already ready. We need to compute two more similarity matrices for drugs using diseases and side effects necessary for the next two drug similarities. In the drug-disease matrix, the methods apply cosine similarity for each pair of drugs. Therefore, its time complexity is $O(m^2n_{di})$. The same happens for the drug-side effect matrix; thus, the complexity of its conversion is $O(m^2n_{se})$. Totally, the conversion for drugs is $O(m^2(n_{di}+n_{se})$. Targets need one extra computation of similarity from diseases. similar to the drug-disease matrix, the complexity of computing similarity among the targets based on their common diseases is $O(n^2n_{di})$. In this paper, *n* is greater than *m*, and the complexity of the similarity computation is $O\left( e_{emb}\left( (m+n)T_{e}\right) \right)$, and $m<n$; thus, it is $O\left( e_{emb}nT_{e}\right)$. IEDTI computes embeddings of drugs and targets. These secondary representations have a time complexity of $O(m^2n_{di})$.

Both models have a similar predictive module, and their complexity to evaluate all targets and all drugs is $O\left( e_{p}mnT_{p}\right)$. Their difference is in $T_{p}$, which IDETI needs lower time and space complexity than the DEDTI.

It is notable that IEDTI with three DNN modules (two for embedding vectors’ production and one module for prediction) contains all the steps of embedding preparation and prediction, while the state-of-the-art methods use the available embeddings (e.g., TransDTI) or have higher complexity (IMCHGAN).

### Molecular docking analysis

Structure-based Molecular docking is a virtual alternative to costly and time-consuming laboratory experiments to find the “best-fit” orientation of a drug to a particular target. Thus, we used this technique to rationalize the interaction potential between Chlorzoxazone-PTGS2 and Tetrabenazine-ADORA1 as two novel predicted drug-target pairs. To this end, crystal structures of ADORA1 (PDB 5n2s) and PTGS2 (PDB 3QMO) were obtained from the RCSB PDB protein data bank^37^. Also, the 3D-SDF structures of the tetrabenazine and chlorzoxazone were downloaded from the NCBI PubChem^38^. The native ligand, HEATM, and other solvent molecules in both protein structures were removed using discovery studio, and the steepest descent method was utilized for energy minimization. Then, the Swiss PDB Viewer (SPDBV) tool^39^ was used to acquire the most stable conformation of proteins. Eventually, the final stages of protein preparation, including the addition of polar hydrogens and Kollman charges, were done using the Autodock tools (ADT). The preparation of ligands was performed by the addition of polar hydrogens and gasteiger charges. Also, root detection and choosing torsions from the torsion tree were done to rotate all the rotatable bonds. In order to determine the “active site” in the bonding position of ADORA1, the crystal structure of stabilized ADORA1in complex with PSB36 at 3.3A was visualized using the LIGPLOT+ tool^40^. The obtained pattern shows the His 1356, Trp 1352, Leu 1355, Met 1285, Asn 1359, Thr 1375, Glu 1277, Thr 1362, Phe 1276, Val 1192, Ile 1174, Ile 1379 and Ala 1196 are the most important amino acids involved in forming this complex. Furthermore, the X-ray crystal structure of NS-398 bound to cyclooxygenase-2 was analyzed. Arg 120, Val 523, Ala 527, Val 349, Ser 530, Tyr 385, Trp 387, Gly 526, Leu 352, Met 522, Phe 518, and Ser 353 were determined as most participant residues for establishing the above-mentioned complex. To define docking space, we generated the grid box for each target protein. For ADORA1 the grid box values are x-center = 103.962, y-center = 128.898, z-center = 44.237, and x-points = 54, y-points = 48, and z-points = 58. For PTGS2, the center grid box is defined with 40.049, 51.442, and 69.613 as X-, Y- and Z-, respectively, and the grid points were 56, 60, and 63 in X-, Y-, and Z-coordinates. Also, the grid point spacing was set to 0.375 angstroms for both of them. Finally, docking studies were performed by AutoDock 4.2 using the Lamarckian genetic algorithm.

## Results

### Overview of DEDTI and IEDTI

In order to narrow the experimental space required to discover a novel therapeutic agent, this study proposes two innovative computational models called IEDTI and DEDTI. They can assist in identifying new DTIs by incorporating heterogeneous information on drugs and targets. IEDTI and DEDTI scenarios take advantage of the drug-target interactions as the prediction label. As an overview (Figs. 1 and 2) represent IEDTI and DEDTI, respectively. Both models extract four types of similarities between drugs and three types of similarities for targets. Both scenarios manipulate the accumulative version of drugs and targets as their inputs. IEDTI consists of three CNN modules. The first and second modules generate the embedding vectors of drugs and targets, respectively. Thus, their inputs are feature vectors from the accumulation of similarity matrices, and their outputs are new embedding vectors. To have a meaningful generation of embeddings, a clustering method is applied to the accumulation matrices. The clustering helps to identify labels of drugs and targets. The DNN modules generate similar embedding vectors for inputs with the same label. The third module identifies the interaction of each drug-target pair. Thus, its input is the concatenation of new embedding vectors of drug-target pairs, and its output is a binary value that shows the existence or lack of any interaction. DEDTI, on the other hand, consists of just a single DNN module. The inputs of this module are directly accumulated similarity matrices of each under-examination drug-target pair, and its output is their interaction identifier. The "Methods" section describes both scenarios in detail.

### Comparison of performance with other existing models

**Figure 3:**
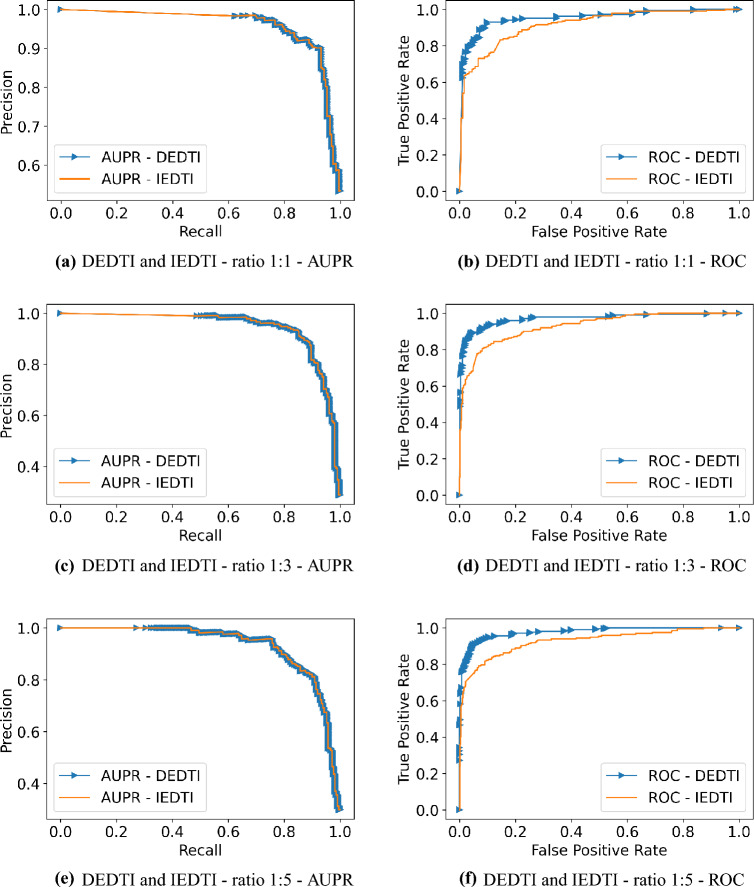
PR and ROC curves of different sampling ratios from DTINet dataset.

The prediction performance of our models was evaluated using a ten-fold cross-validation procedure. We divided the data set into the test and training sets, where $10\%$ of the data set was utilized as the test set, and the remaining $90\%$ was used as the training set. Then, we compared our results with the results of five state-of-the-art methods for DTI prediction, including HIDTI^22^ and NeoDTI^20^, MolTrans^23^, TransDTI^24^, and IMCHGAN^41^. Also, due to data imbalance in positive vs. negative samples of DTI, we report the results with positive to negative ratios of 1:3, and 1:5, as common in the literature^22^. Tables 2 and 3 illustrate the results for these two sampling ratios, respectively. We compare the results based on AUC-ROC and AUPR, precision, recall, F1-score, and MCC. AUPR, F1-score, and MCC especially are insightful when there exists a ratio imbalance among positive and negative samples. IEDTI has a higher AUC-ROC in comparison with the HIDTI models and NeoDTI. The HIDTI-simple format has a higher AUPR in 1:3 and 1:5 ratios than IEDTI. However, the standard deviation of HIDTI models and NeoDTI is much higher than the IEDTI. In other words, IEDTI has lower fluctuations in seeing diverse folds. More importantly, as the table shows, DEDTI provides the best AUPR and AUC-ROC across all methods with minor fluctuations through all ratios and in both metrics. The results show that IEDTI and DEDETI, especially the latter, perform well in the prediction of DTIs. Figures 3a–f show the ROC and PR plots of IEDTI and DEDTI for all ratios 1:1, 1:3, and 1:5. It is worth mentioning that the same happens for IEDTI and DEDTI methods for the ratio of 1:10.

**Table 2:** Prediction performance comparison for a 1:3 ratio of positive and negative interactions in DTINet dataset.

Methods	AUC-ROC	AUPR	Precision	Recall	F1	MCC
HIDTI-simple format^22^	0.879	0.903±0.02	0.732	0.708	0.716	–
HIDTI (all+PDIS)^22^	0.901	0.756±0.03	0.799	0.781	0.787	–
NeoDTI^20^	0.809	0.756±0.03	0.622	0.646	0.629	–
IMCHGAN	0.956	0.920	0.892	**0.859**	**0.875**	**0.835**
IEDTI	0.919 ±0.0001	0.843 ±0.0005	0.819 ±0.0021	0.739 ±0.0011	0.776 ±0.0002	0.709 ±0.0003
DEDTI	**0.961±0.0001**	**0.925±0.0002**	**0.899±0.0011**	0.845 ±0.0002	0.871 ±0.0002	**0.835±0.0005**

The best performance values are in bold.

**Table 3:** Prediction performance comparison for a 1:5 ratio of positive and negative interactions in DTINet dataset.

Methods	AUC-ROC	AUPR	Precision	Recall	F1	MCC
HIDTI-simple format^22^	0.853	0.818±0.04	0.670	0.607	0.630	–
HIDTI (all+PDIS)^22^	0.904	0.629±0.05	0.770	0.717	0.740	–
NeoDTI^20^	0.841	0.604±0.07	0.497	0.583	0.605	–
IMCHGAN	0.959	**0.907**	0.873	**0.845**	**0.858**	**0.829**
IEDTI	0.916 ±0.0001	0.789 ±0.0003	0.781 ±0.0025	0.698 ±0.0012	0.736 ±0.0005	0.689 ±0.0008
DEDTI	**0.961±0.0001**	0.898 ±0.0005	**0.880±0.0024**	0.813 ±0.0021	0.844±0.0005	0.816 ±0.0007

The best performance values are in bold.

**Figure 4:**
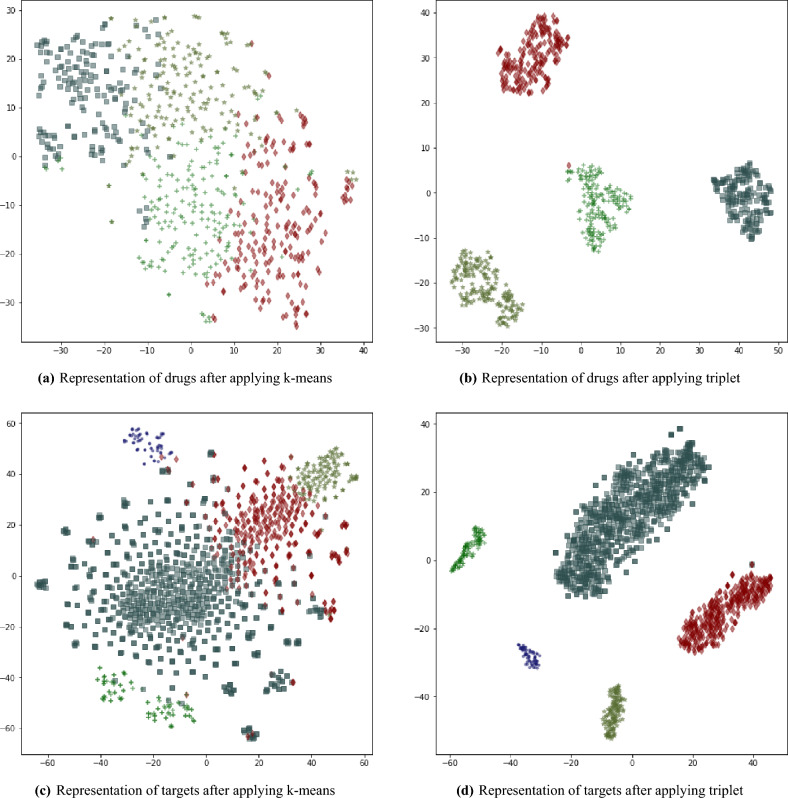
The t-NSE representations of Drugs and targets in DTINet dataset. The left figures show the representations of drugs and targets after applying the k-means. As the figures show, the classes have not been disjointed completely; however, by applying the triplet modules, both drugs, and targets are disjointed completely (right figures).

### External validation on gold-standard datasets

**Figure 5:**
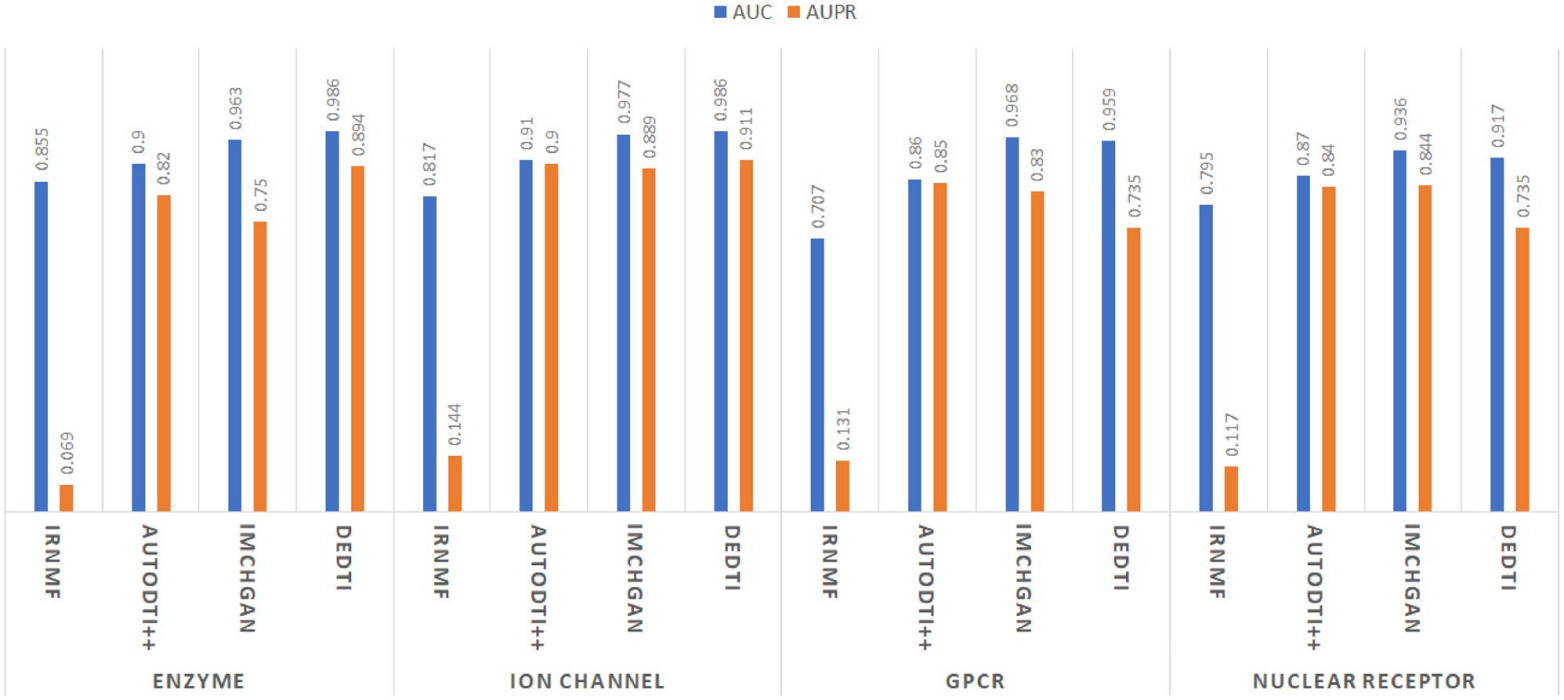
Performance comparison among DEDTI with IRNMF, AutoDTI++, and IMCHGAN on the gold-standard datasets^31^. AUC-ROC and AUPR bar charts.

We apply the DEDTI, IMCHGAN, AutoDTI++, and IRNMF on gold-standard datasets^31^ (Enzyme, Ion Channel, GPCR, and Nuclear Receptor datasets). Their AUC-ROC and AUPR bar charts are shown in Fig. 5. As the results show IMCHGAN and DEDTI have a tight competition on gold-standard datasets. While IMCHGAN has the highest AUC-ROC in GPCR and Nuclear Receptor, the DEDTI has the highest AUC-ROC in Enzyme and Ion Channel datasets. In addition, the bar charts diagram shows that the DEDTI has the highest AUPR in three out of four benchmarks. Moreover, Table 4 presents the comparison of DEDTI, TransDTI, MolTrans, TransforerCPI, DeepConvDTI, and DeepDTA on gold-standard datasets. The DEDTI is the winner in all cases except two.

**Table 4:** External validation on gold-standard datasets^31^ illustrated the effectiveness of DEDTI against state-of-the-art competing methods.

Validation Set	Metric	DeepDTA	DeepConvDTI	TransforerCPI	MolTrans	TransDTI	DEDTI
GPCR	Accuracy	0.73	0.68	0.69	0.72	0.77	**0.96**
	MCC	0.58	0.67	0.56	0.59	**0.69**	**0.69**
	F1 score	0.63	0.68	0.59	0.62	0.60	**0.70**
	Sensitivity	0.67	0.67	0.54	0.55	0.58	**0.99**
	Specificity	0.78	0.72	0.80	0.84	**0.85**	0.68
Enzyme	Accuracy	0.62	0.57	0.67	0.69	0.75	**0.99**
	MCC	0.47	0.41	0.57	0.55	0.59	**0.87**
	F1 score	0.59	0.56	0.64	0.65	0.70	**0.87**
	Sensitivity	0.53	0.53	0.59	0.61	0.68	**1.0**
	Specificity	0.69	0.64	0.77	0.77	0.81	**0.82**
Ion Channel	Accuracy	0.67	0.59	0.62	0.65	0.63	**0.98**
	MCC	0.49	0.38	0.49	0.45	0.48	**0.84**
	F1 score	0.62	0.56	0.55	0.56	0.59	**0.84**
	Sensitivity	0.60	0.52	0.50	0.51	0.57	**0.99**
	Specificity	0.72	0.67	0.72	0.76	0.70	**0.84**
Nuclear Receptors	Accuracy	0.58	0.61	0.64	0.71	0.74	**0.96**
	MCC	0.35	0.43	0.48	0.52	0.55	**0.72**
	F1 score	0.53	0.56	0.50	0.58	0.68	**0.73**
	Sensitivity	0.50	0.50	0.45	0.52	0.63	**0.98**
	Specificity	0.67	0.72	0.77	0.83	**0.84**	0.71

Significant values are in bold.

### DEDTI predicts novel interactions

**Figure 6:**
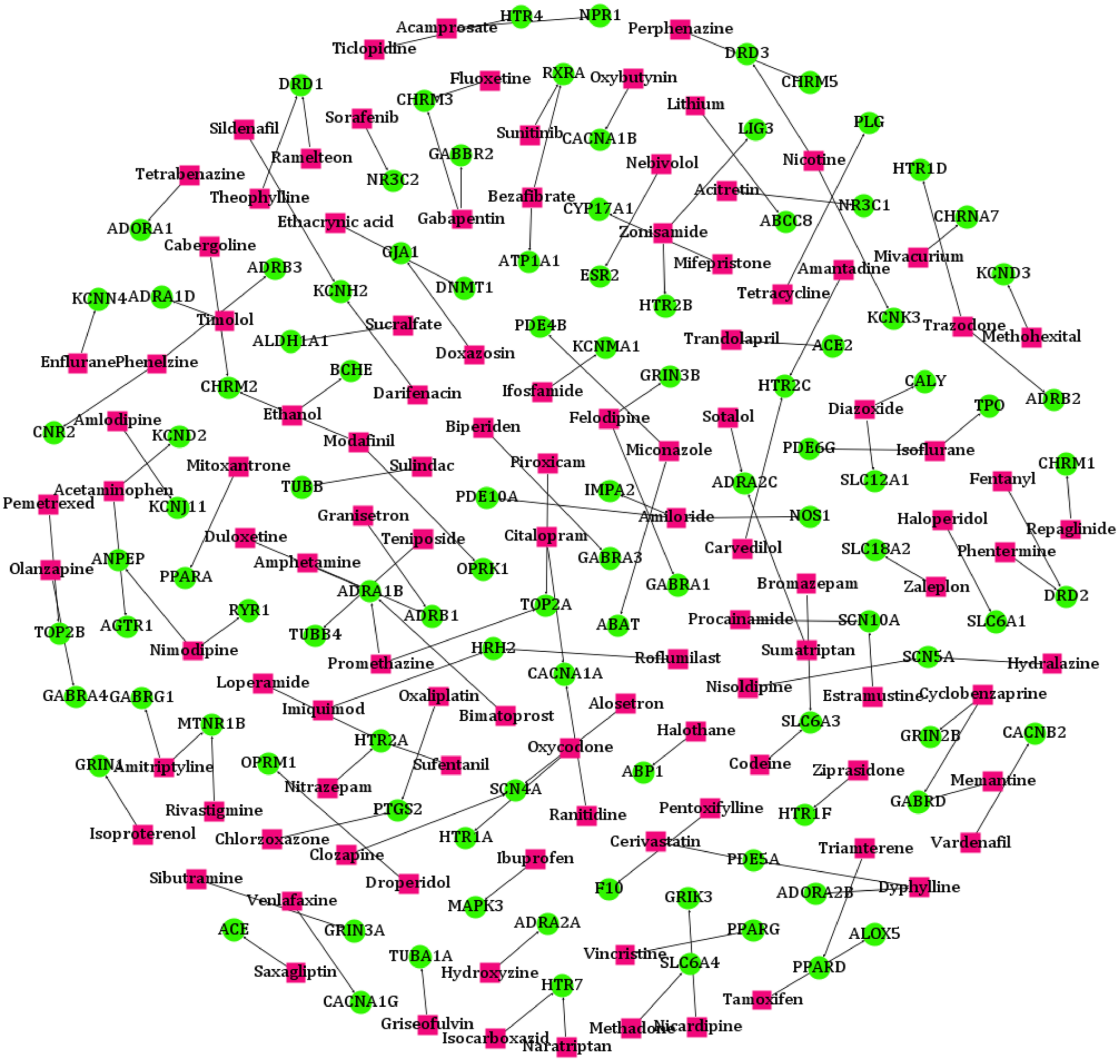
Visualization of the top 126 DTIs predicted by DEDTI. Targets are shown in green circles, and drugs are shown in pink boxes. Drug-target novel interactions are marked by black edges.

Our model uses the information from accumulative similarities to predict the novel interactions among drugs and targets (Supplementary Data 1). We selected DTIs with a prediction score of not less than 0.9 as the top-ranked suggestions of DEDTI. Among the 126 top-ranked predictions (Fig. 6), we figured out that many of them are verifiable with scientific evidence from the literature. For instance, our prediction list shows the interaction between fentanyl and the D2 dopamine receptor (DRD2), and this prediction can be supported by previous studies^42^.

However, among the list of top 126 predictions from DEDTI, there are some novel interactions with less attention in the literature. For example, two of these interactions are tetrabenazine- adenosine receptor A1 (ADORA1) and chlorzoxazone- prostaglandin-endoperoxide synthase 2 (PTGS2). Adenosine receptor A1 along with four other receptors are forming a defined subgroup of G protein-coupled receptors^43^. This protein is spread all over the human body and regulates renal function^44^. Moreover, recent studies show that the knockdown of ADORA1 in the human melanoma cell lines significantly suppresses cell proliferation, and this suppression leads to an antitumor effect^45^. Although, according to the KEGG database^46^, there are 25 approved drugs affecting ADORA1, the predicted drug by DEDTI (tetrabenazine) is not mentioned in this list. Tetrabenazine has been known as a dopamine-depleting agent developed for the treatment of schizophrenia. Additionally, many studies demonstrated this drug could be effective in the treatment of psychotic disorders and hyperkinetic movement disorders^47^. Prostaglandin-endoperoxide synthase 2 (PTGS2), also known as cyclooxygenase 2 (COX-2), is responsible for prostaglandin production and contributes to early pregnancy^48^. Furthermore, numerous studies have been reported on the role of PTGS2 in the pathogenesis of many diseases, such as inflammation, cardiovascular, gastrointestinal, and colorectal cancer^49^. Non-steroidal anti-inflammatory drugs (NSAIDs) are commonly used as an inhibitor for this enzyme^50^. Chlorzoxazone is an FDA-approved muscle relaxant, which was also predicted by DEDTI as a potential drug for interacting with PTGS2. In spite of the availability of approved drugs for these two above-mentioned targets, identifying a novel drug from existing approved drugs is always considerable. Therefore, it would be fascinating to check whether the predicted interactions between these two drugs and targets can be further validated.

### Molecular docking suggests binding affinities

**Figure 7:**
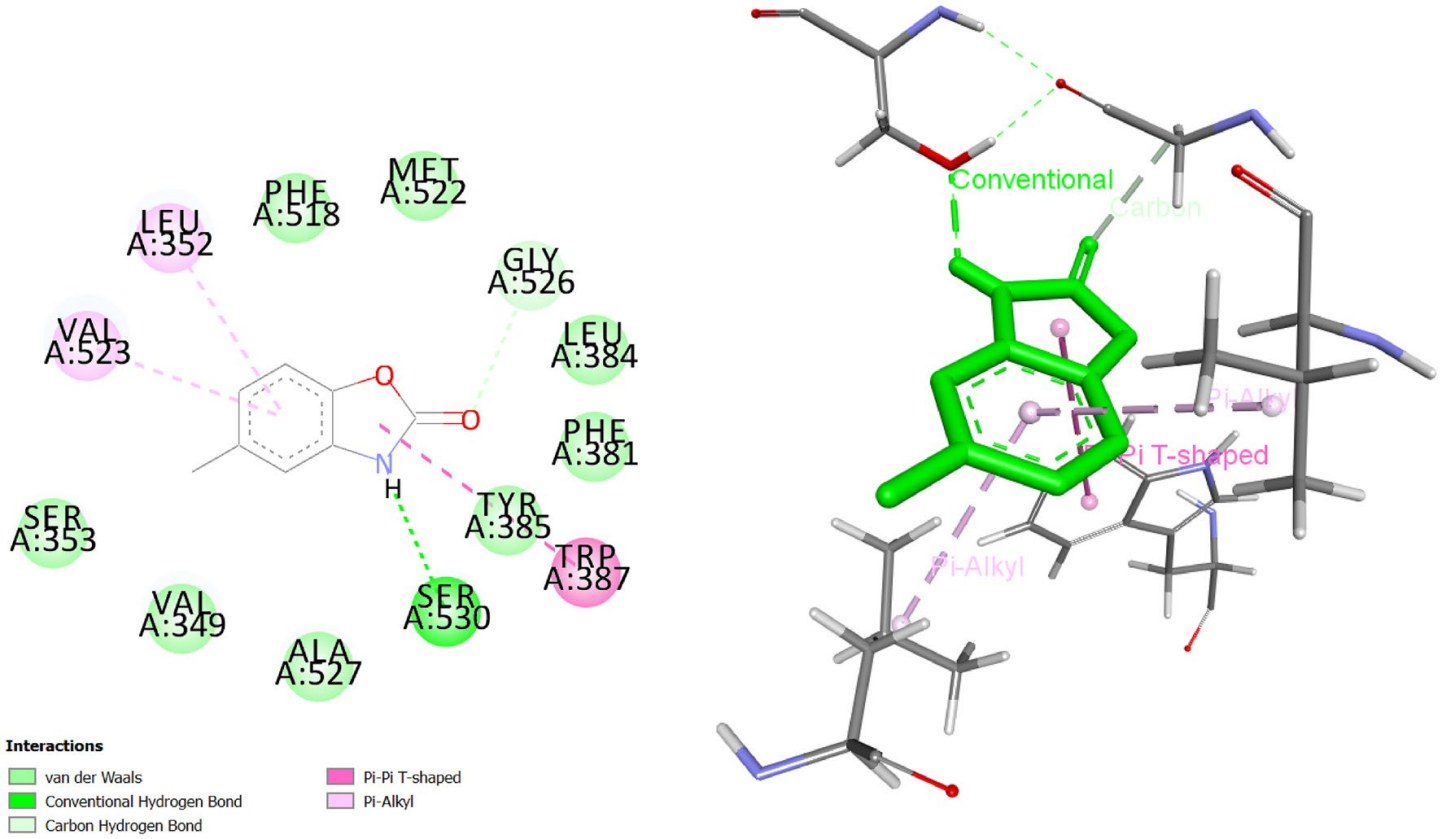
2D and 3D representations of the docked pose for the predicted interaction between chlorzoxazone and PTGS2. Hydrogen bonds are represented by the green dashed lines.

**Figure 8:**
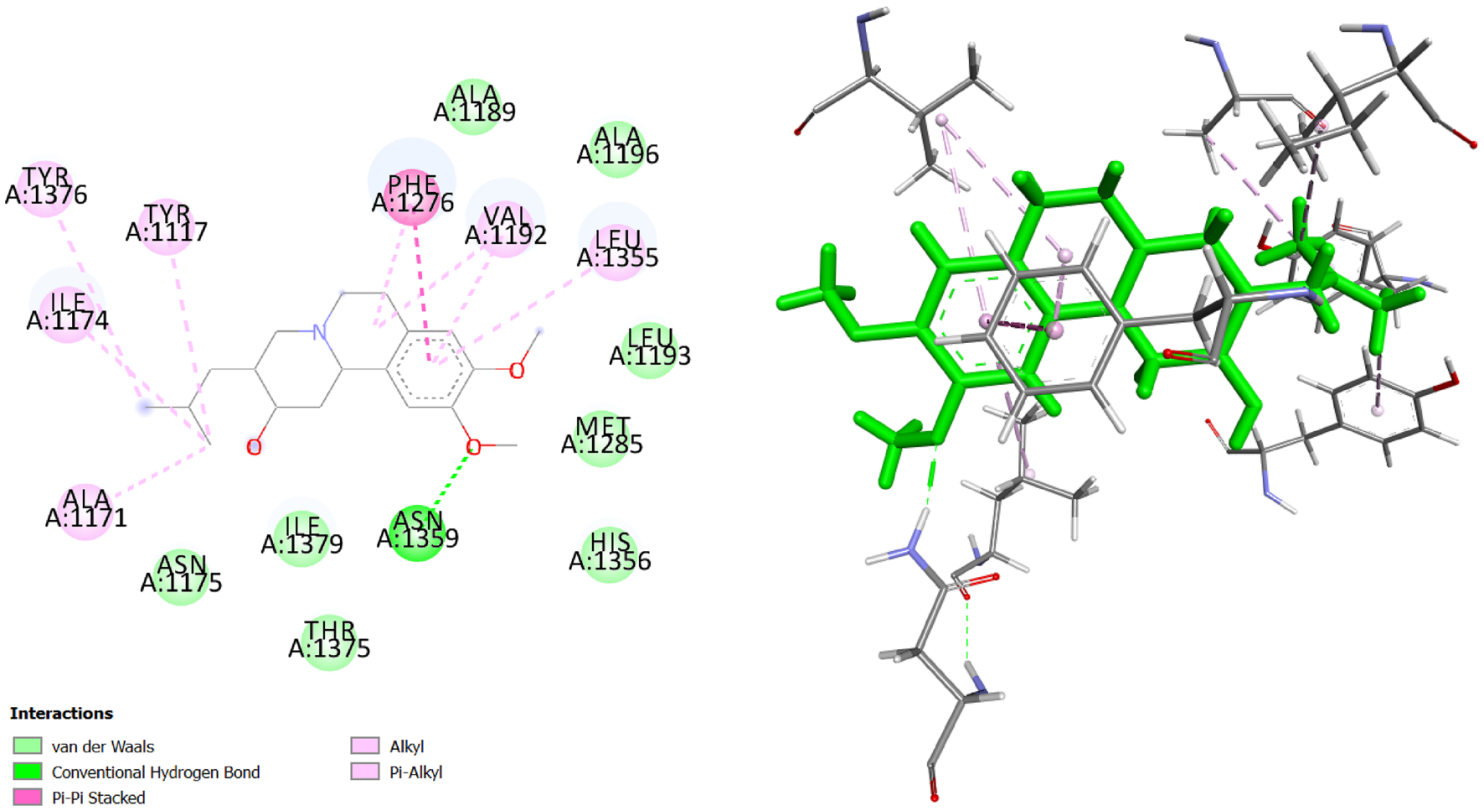
2D and 3D representations of the docked pose for the predicted interaction between tetrabenazine and ADORA1. Hydrogen bonds are represented by the green dashed lines.

Molecular docking studies were done to analyze the possible interactions between the chlorzoxazone, and tetrabenazine complexed with PTGS2 and ADORA1, respectively. The obtained conformations were clustered based on the conformational similarities and root-mean-square positional deviation (RMSD)^51^. Then, the best pose with the lowest binding energy ($\Delta G$) was selected for each target. For the purpose of investigating the intermolecular interaction forces, the docking results were visualized using Biovia Discovery Studio Visualizer^52^. The binding free energies of chlorzoxazone and tetrabenazine complexed with PTGS2 and ADORA1 are shown in Table 5. Both predicted drugs bind to their target with acceptable binding affinities and in a correct position. Chlorzoxazone binds to PTGS2 by forming a hydrogen bond with Ser 530 and other interactions with Val 523, Leu352, Phe 518, Met 522, Gly 526, Lue 384, Phe 381, Tyr 385, Trp 387, Ala 527, Val 349 and Ser 353. Figure 7 shows its 3D and 2D representations. As Fig. 8 shows, the complex of tetrabenazine-ADORA1 is formed by an intermediate of a hydrogen interaction between the drug and Asn 1359. In addition, other amino acids such as Ala 1171, Ile 1174, Tyr 1376, Tyr 1117, Phe 1276, Val 1192, and Leu 1355 were also involved in forming this drug-protein complex.

**Table 5:** Docking results for two novel interactions (chlorzoxazone-PTGS2 and tetrabenazine-ADORA1) predicted by DEDTI.

Compound Name	PUBCHEM CID	Molecular Formula	Docking Score (kcal/mol)	
			PTGS2	ADORA1
Chlorzoxazone	2733	$C_7 H_4 ClNO_2$	−6.21	–
Tetrabenazine	6018	$$C_{19} H_{27} N_3 $$	–	−7.51

### Statistical analysis

We performed the t-test with an error level of $5\%$ to check the significance of differences in results on three methods of IEDTI, DEDTI, and IMCHGAN on all datasets. Here we report the results on DTI with a negative sampling ratio of 1:1, DTI with a negative sampling ratio of 1:3, and all gold-standard datasets. In all cases, the statistical analysis was below the error level except the case of comparing DEDTI and IMCHGAN on the DTI dataset with a negative sampling ratio of 1:1. In other words, in all cases, DEDTI is significantly better than the other methods. The exception happens for the ratio 1:3, in which the DEDTI and IMCHGAN perform equally. Table 6 shows the results of the *p*-value.

**Table 6:** Results of the *p*-value test between DEDTI and other methods.

	IMCHGAN	IEDTI
*p*-value	0.00424	7.22E−07

## Discussion and conclusion

We have introduced two methods, IEDTI and DEDTI, which both need the drug-target interactions not as input feature information but as labels for DTIs prediction. In other words, our methods are inductive, which contrasts with NeoDTI^20^. NeoDTI uses drug-target information in feature space, which is quite common in graph neural network methods. More importantly, both train and test samples are visible in the method’s training phase, which makes this method transductive. Transductive methods are not suitable for prediction.

IEDTI and DEDTI utilize DNN modules for their missions. the former uses three modules (two for the production of embeddings and one for prediction and the latter uses one module (the prediction module). besides the number of modules, both of them have a lower computational complexity in comparison to state-of-the-art methods, e.g., HIDTI, NeoDTI, and IMCHGAN. Additionally, IEDTI acquires meaningful embeddings directly instead of using available and ready-to-use embeddings.

On the other hand, IEDTI, like methods from the literature such as NeoDTI and HIDTI, takes advantage of transforming the original feature space to a new corresponding embedding space. It aims to have a meaningful representation of data and a lower computational overhead for the prediction. We show this in the complexity analysis in the Method section. However, such transformations depend on the conversion method and the labeled data. In many cases, data clustering does not return a suitable value. DEDTI presents that more straightforward methods without the extra overhead of embedding conversion outperform better in DTI prediction. It is necessary to have better methods for embedding conversions.

Moreover, methods need to be inductive to be capable of predicting DTIs. Based on Occam’s razor, the more straightforward method is the best choice for the data. Again DEDTI gives an insightful representation of this idea. Information for DTI, i.e., drug-target interactions, drug-drug interaction, drug-drug similarity, drug-side effect associations, drug-disease associations, target-target interactions, target-disease interactions, similarities of targets. Another important observation from this work is the advantages of summing up similar matrices instead of concatenating them. Converting the information matrices to the similarity matrices makes their dimension equal, and this conversion provides the capability of summing the information.

The summation of similairty matrices has a smaller feature space than the concatenation. For example, each drug vector has a size of 708 compared to other methods with feature vector’s length greater than thousands. In addition, the concise feature space avoids the sparse representation of the feature vectors. In other words, each drug sample has a denser representation, making them more meaningful.

The denser representation is another reason why DEDTI has the best performance across all methods. Notably, in addition to the deep prediction network, DEDTI includes the summed-up similarity vectors as the feature representation of both the drug and target. Improving the way of feature embeddings and ameliorating the inductive, predictive method are elixirs of DTI prediction.

## Supplementary Information

Supplementary Information.

## Data Availability

The datasets generated and/or analyzed during the current study are available in the IEDTI-DEDTI repository, github.com/BioinformaticsIASBS/IEDTI-DEDTI.
